# Amyand's Hernia With Mixed Content, Cecal Perforation, and Fournier's Gangrene: A Case Report and Literature Review

**DOI:** 10.7759/cureus.113688

**Published:** 2026-07-30

**Authors:** Arturo A Flores Morales, Abelardo Flores Morales, Ricardo Y Topete De Robles, Antonio Gael Ramos Ramirez

**Affiliations:** 1 General Surgery, Hospital Civil de Tepic "Dr. Antonio González Guevara", Tepic, MEX; 2 Cardiology, Centro Médico Nacional Siglo XXI, Mexico City, MEX; 3 Surgery, Hospital Civil Tepic, Tepic, MEX; 4 Surgery, Servicios de Salud del Instituto Mexicano del Seguro Social para el Bienestar, Tepic, MEX

**Keywords:** amyand's hernia, bowel obstruction, cecal perforation, emergency surgery, fournier's gangrene, inguinoscrotal hernia

## Abstract

Amyand's hernia is defined by the presence of the vermiform appendix within an inguinal hernia sac and occurs in fewer than 1% of all inguinal hernias. When the appendix is inflamed or perforated, the incidence decreases to 0.1%, and when additional intra-abdominal organs are involved, the condition becomes exceptionally rare. We present the case of a 62-year-old man with a 10-year history of an untreated right-sided inguinoscrotal hernia who presented to the emergency department with a five-day history of progressive scrotal swelling, discoloration, pain, and inability to defecate or tolerate oral intake. Examination revealed a 17 × 10 cm inguinoscrotal mass with violaceous skin discoloration and visible necrosis at the scrotal base. Plain abdominal radiographs showed air-fluid levels consistent with small bowel obstruction. Emergency laparotomy confirmed a Losanoff-Basson type 4 Amyand's hernia with right-sided mixed hernial contents extending 370 cm from the angle of Treitz into the scrotum, including the cecum, appendix, and terminal ileum, with cecal perforation, appendiceal compromise, fecaloid spillage, and Fournier's gangrene of the scrotum​​​, with approximately 30 cm of small bowel.​​

Surgical management included resection of the small bowel at 340 cm from the angle of Treitz with a colonic pouch, terminal ileum ileostomy, right orchiectomy, scrotal debridement with repeated surgical washouts, and primary hernia defect repair without mesh. The patient underwent five additional surgical washouts during a 33-day hospitalization, followed by intestinal transit restoration during outpatient follow-up. To our knowledge, this case represents one of the most anatomically extensive right-sided Amyand's hernias with simultaneous cecal perforation and Fournier's gangrene reported in the indexed literature. Early surgical recognition and aggressive multivisceral management were the determining factors in the patient's survival.

## Introduction

Amyand's hernia, named after Claudius Amyand, who performed the first recorded appendectomy in 1735 on an appendix found within an inguinal hernia sac, is an uncommon type of inguinal hernia, occurring in fewer than 1% of all inguinal hernias. In the largest systematic review to date, covering 442 patients across 231 studies, 91% of cases occurred in males, and 9.5% involved a right-sided presentation, making right-sided Amyand's hernia the rarest presentation of inguinoscrotal hernias. Acute appendicitis within the hernial sac occurs in only 0.1% of inguinal hernias, and the simultaneous presence of additional intra-abdominal structures as hernial content alongside a perforated appendix is rarer still.

Losanoff and Basson proposed a four-type classification in 2008 that remains the most widely used framework for surgical decision-making in this condition. Type 1 describes a normal appendix within the sac; type 2, an inflamed appendix without abdominal involvement; type 3, appendicitis with associated abdominal pathology; and type 4, any Amyand's hernia occurring alongside unrelated abdominal pathology. Type 4 carries the highest surgical complexity and has been associated with mortality rates between 14% and 30% when complicated by peritonitis or sepsis.

Fournier's gangrene is a polymicrobial necrotizing fasciitis of the perineum and genitalia with an overall mortality rate exceeding 20% in most series, rising sharply when diagnosis is delayed or when the source control is incomplete. Its concurrence with Amyand's hernia, typically driven by perforated appendicitis seeding the scrotal compartment, has been described only in isolated case reports. The present case is distinguished​​​​​​ from all prior reports by a combination of three features that have not been documented together: right-sided Amyand's hernia, hernial contents extending 370 cm from the angle of Treitz into the scrotum with cecal perforation as the primary driver, and concurrent Fournier's gangrene requiring staged multivisceral resection and orchiectomy.

Surgical planning is further complicated by the degree of contamination at the time of intervention, which determines whether primary repair, mesh avoidance, bowel resection, or stoma formation is required. These decisions frequently have to be made intraoperatively without the benefit of preoperative staging.

We report this case to document the surgical anatomy, operative decision-making, and staged postoperative management of what appears to be one of the most anatomically extensive presentations of type 4 Amyand's hernia with Fournier's gangrene reported​​​​ in the published literature, treated successfully at a resource-limited public hospital in western Mexico.

This case was presented as a research poster at the Asociacion Mexicana de Cirugia General (AMCG) Annual Congress, 2025.

## Case presentation

A 62-year-old male agricultural worker with a history of occasional tobacco and alcohol use and a prior diagnosis of tuberculosis, treated five years before presentation, was brought by ambulance to the emergency department. He reported a 10-year history of a right inguinal hernia that had been managed conservatively, as the patient did not want to undergo surgical correction. On arrival, his most prominent complaint was a five-day history of progressive right inguinoscrotal swelling with skin discoloration, intense abdominal pain rated 3 out of 10 on the visual analogue scale at the onset, increasing to 7 out of 10, constipation, inability to tolerate oral intake, nausea, and vomiting that had started three days earlier. He denied fever, urinary symptoms, hematemesis, or melena. No anticoagulant or antiplatelet medications were documented.

Physical examination

On arrival at the emergency department, his vital signs were as follows: blood pressure, 104/72 mmHg; heart rate, 104 bpm; respiratory rate, 19 breaths/minute; temperature, 36.2°C; and oxygen saturation, 96% on room air.

Neurological status was assessed as a Glasgow Coma Scale score of 15. Head and neck examination was unremarkable. Cardiopulmonary examination showed no acute compromise. The abdomen was soft, with diffuse tenderness on deep palpation, particularly in the epigastric region, right iliac fossa, and left iliac fossa, with signs consistent with peritoneal irritation. Bowel sounds were diminished.

**Figure 1:**
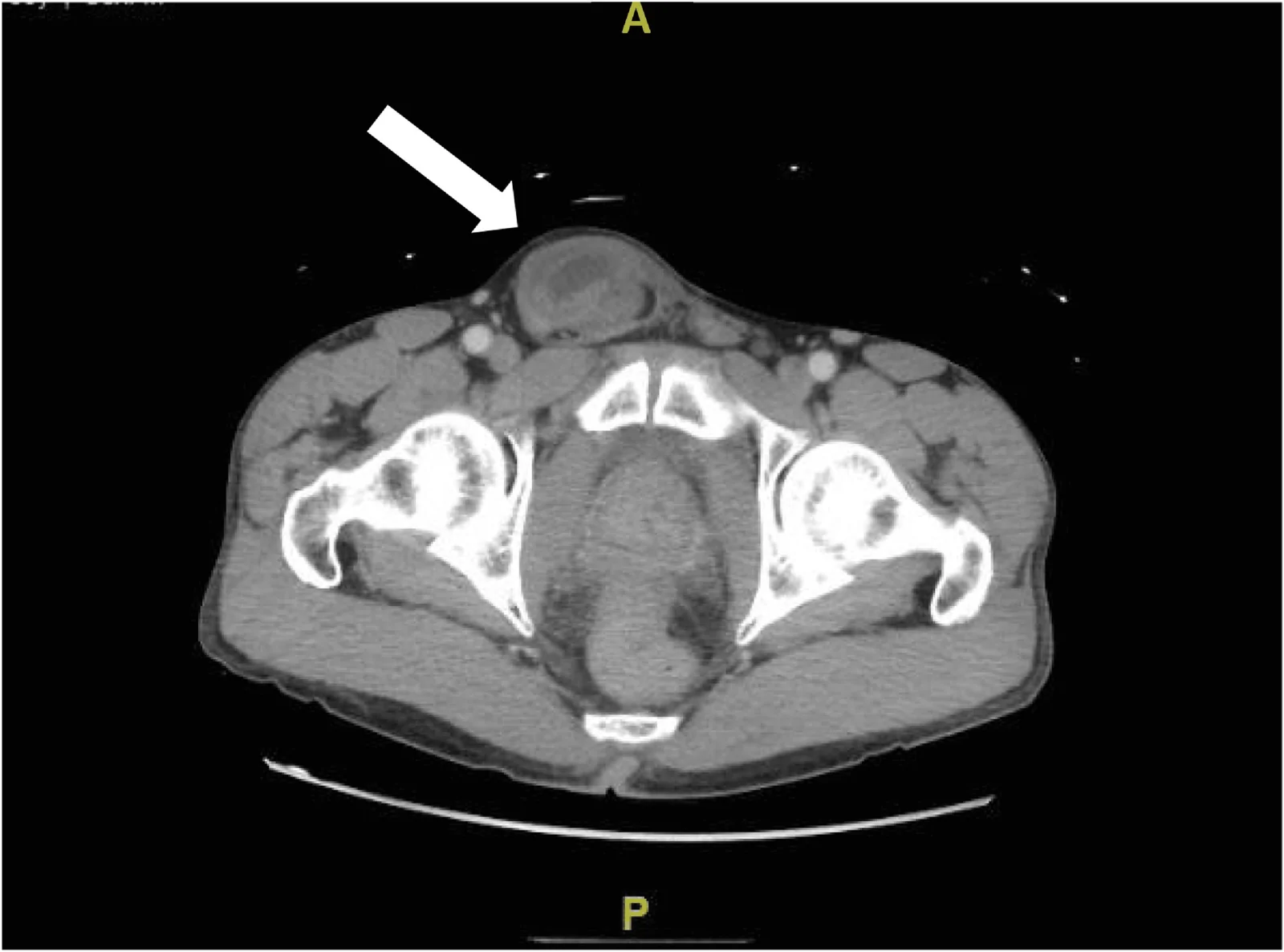
Illustrative CT scan of right inguinoscrotal hernia (Amyand's hernia)

The right inguinoscrotal region showed a cylindrical, non-reducible mass measuring approximately 17 × 10 cm, with visible obstruction of the inguinal canal. The overlying skin was dark violaceous and indurated, with frank necrosis at the scrotal base, whereas the left hemiscrotum appeared uninvolved. The lower extremities were hypotrophic but intact (Figure 1).

Laboratory findings

Initial serum laboratory results on hospital day 1 are presented in Table 1. Relevant findings included leukocytosis of 20.7 × 10³/µL with 89% neutrophils, hemoglobin of 17.8 g/dL with a hematocrit of 55.6% (consistent with hemoconcentration), creatinine of 2.4 mg/dL, sodium of 125 mEq/L, chloride of 75 mEq/L, total bilirubin of 3.38 mg/dL with direct bilirubin of 2.99 mg/dL, AST of 180 U/L, ALT of 67 U/L, albumin of 2.9 g/dL, an INR of 1.31, and a blood glucose level of 142.4 mg/dL. The patient's blood group was O positive. Serial laboratory values over the 33-day hospitalization showed gradual normalization of renal and hepatic markers, with leukocytosis persisting throughout the early postoperative period.

**Table 1:** Laboratory findings on admission to the emergency department

Parameters	Value (July 29)	Reference range
Hemoglobin (g/dL)	17.8	13.5-17.5
Hematocrit (%)	55.6	41-53
Platelets (x10³/µL)	315	150-400
Leukocytes (x10³/µL)	20.7	4.5-11.0
Neutrophils (%)	89	50-70
Lymphocytes (%)	3	20-40
Glucose (mg/dL)	142.4	70-100
Urea (mg/dL)	130.4	15-45
Blood urea nitrogen (BUN) (mg/dL)	60	7-20
Creatinine (mg/dL)	2.4	0.7-1.3
Sodium (mEq/L)	125	136-145
Potassium (mEq/L)	3.3	3.5-5.0
Chloride (mEq/L)	75	98-106
AST (U/L)	180	<40
ALT (U/L)	67	<56
Total bilirubin (mg/dL)	3.38	<1.2
Direct bilirubin (mg/dL)	2.99	<0.3
Albumin (g/dL)	2.9	3.5-5.0
INR	1.31	0.8-1.2
Partial thromboplastin time (PTT) (s)	30.6	25-35
Blood group	O+	—

A midline infraumbilical laparotomy was performed. Exploration of the abdominal cavity revealed that the right inguinal hernia sac contained mixed intestinal contents, including the cecum, appendix, and terminal ileum. Approximately 30 cm of intestine had herniated into the scrotum, extending approximately 370 cm from the angle of Treitz. The cecum showed a frank perforation measuring approximately 1 cm with fecaloid spillage. The appendix was compromised. The ileocecal valve and approximately 30 cm of the terminal ileum with its mesentery were also involved. Approximately 60 cc of fecaloid fluid was present in the abdominal cavity. Diffuse serositis was observed along the entire course of the small bowel. The volume of intra-abdominal inflammatory fluid was estimated at 50 cc. The hernial defect measured approximately 3 cm.

Scrotal exploration confirmed Fournier's gangrene, with indurated gangrenous tissue, fetid odor, involvement of the dartos muscle, and right testicular compromise. Approximately 60 cc of free fecaloid fluid was present within the scrotal cavity.

**Figure 2:**
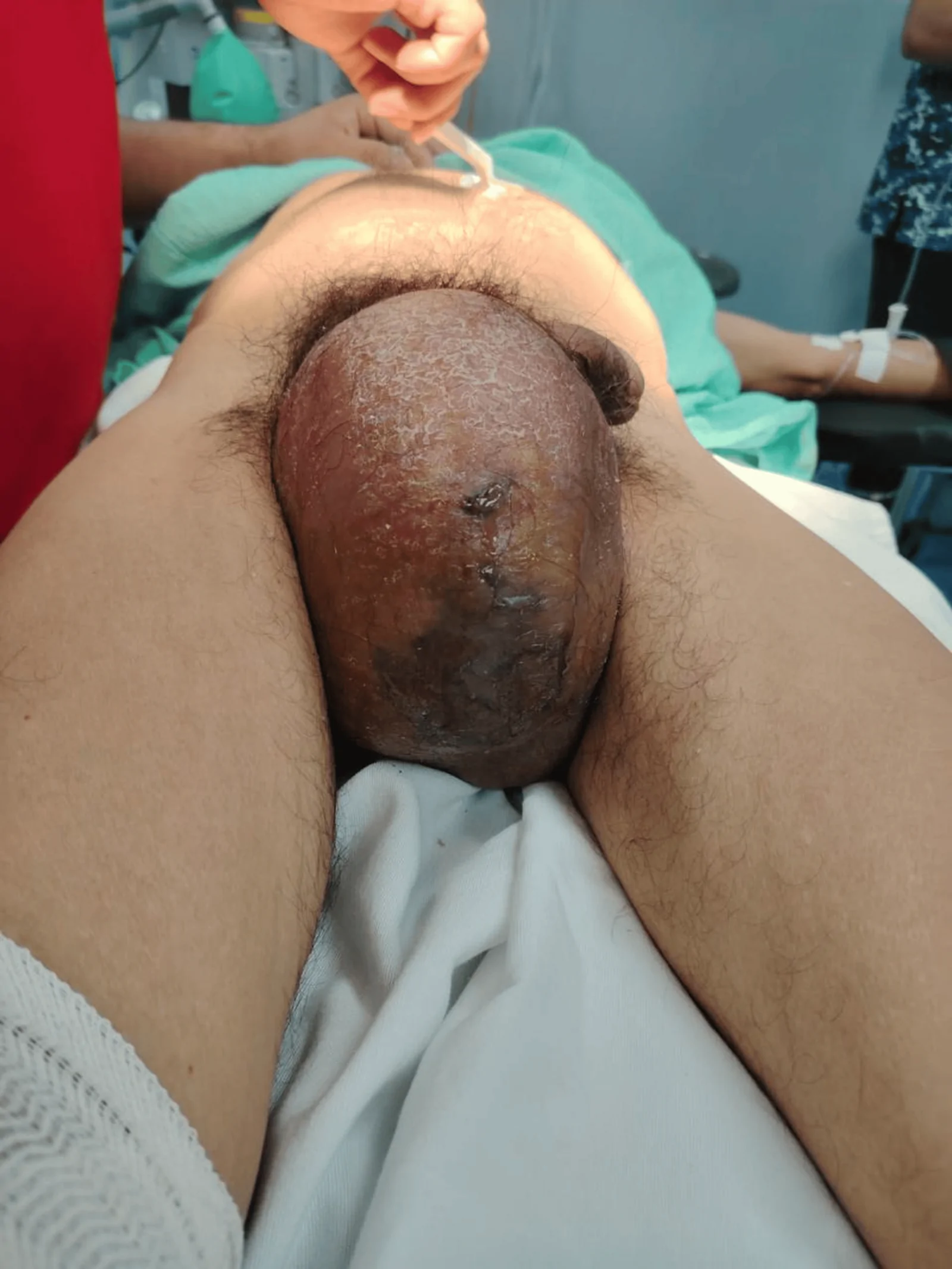
Inguinoscrotal hernia of great dimensions Intraoperative photograph demonstrating the large size of the incarcerated inguinoscrotal hernia and the discoloration of the scrotal skin.

Bowel resection was performed beginning 340 cm from the angle of Treitz through the ascending colon, with mesenteric ligation using 2-0 silk sutures and intestinal clamps. The distal end was closed in a pouch fashion using 2-0 Vicryl. A terminal ileostomy was fashioned at the terminal ileum using the Brooke technique with 2-0 Vicryl sutures. A right orchiectomy was performed because of testicular compromise, with high ligation using 1-0 silk and a transfictive suture. The appendix was included in the resection specimen. Cavity lavage was performed with 5 L of sterile saline solution. The hernial defect was repaired by primary closure using a deep uninterrupted Surgette suture without mesh, as the contaminated surgical field posed a high risk for the use of prosthetic material. Two Penrose drains were placed: one directed to the right paracolic gutter and the other to the pelvic recess. The scrotal wound was debrided with lavage, and Penrose drains were placed in the scrotal region. The abdominal wall was closed with continuous 1-0 Vicryl sutures in the aponeurotic layer and a Surgette suture in the linea alba, with 3-0 Sarnoff nylon sutures in the inguinal area. The surgical time was four hours. Estimated blood loss was 200 mL, and no blood transfusion was required (Figure 2).

**Figure 3:**
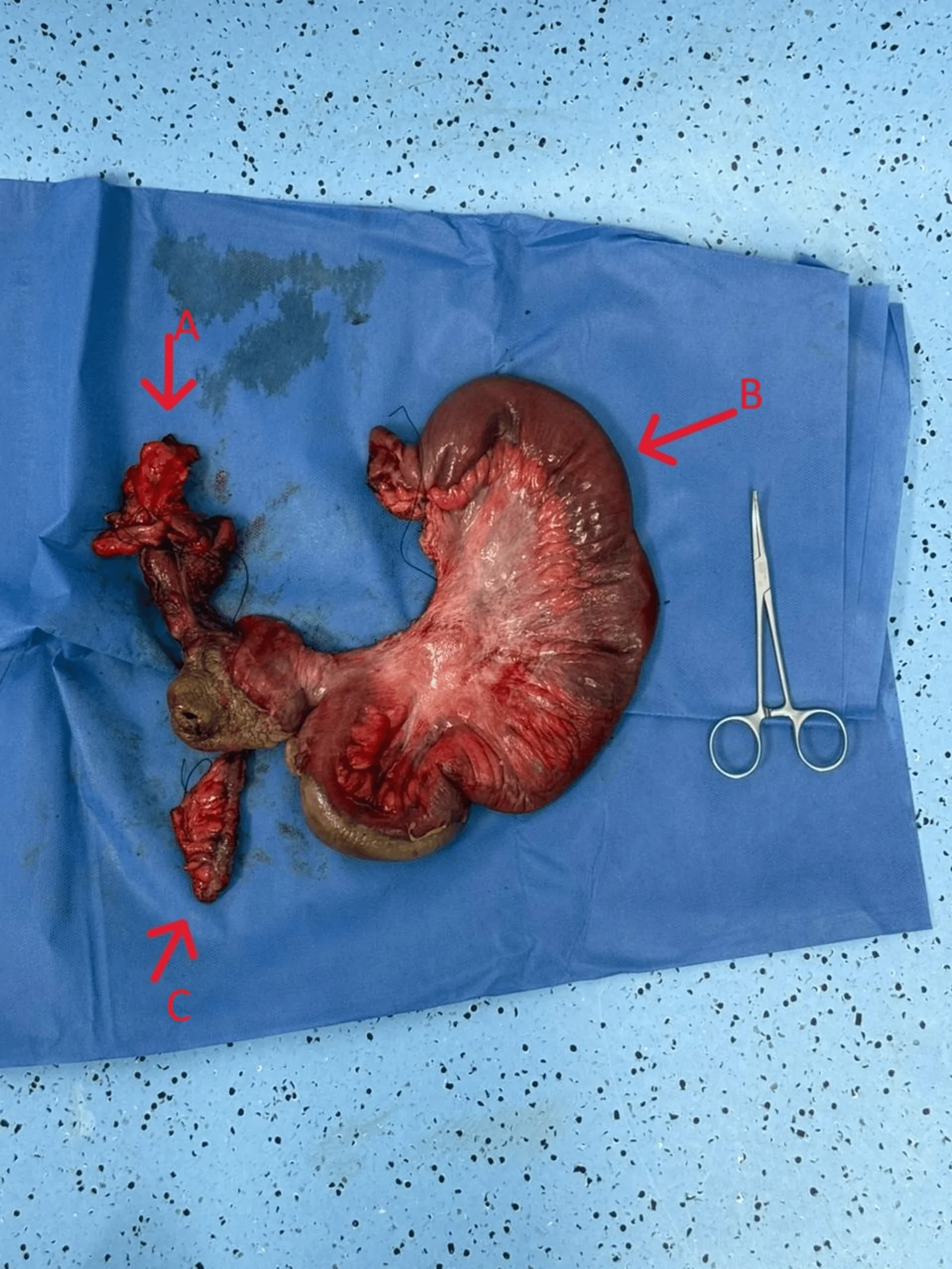
Terminal ileum with the attached appendix, measuring 30 cm in length (A) Appendix. (B) Perforated cecum. (C) Resected small bowel section, measuring approximately 30 cm in length.

The surgical specimen included the ascending colon, ileocecal valve, perforated cecum, appendix, and 30 cm of terminal ileum (Figure 3).

**Figure 4:**
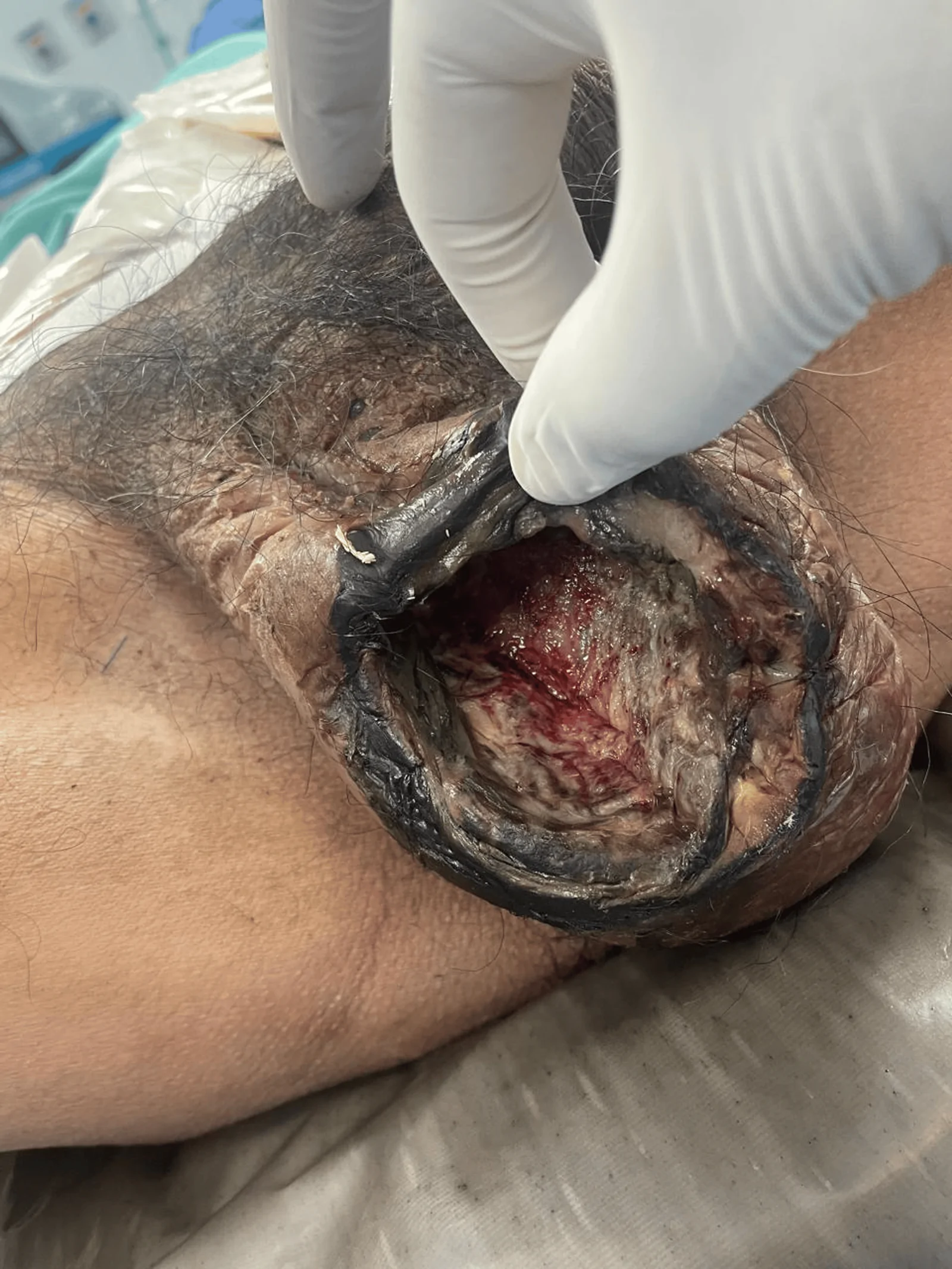
Immediate postoperative appearance of the scrotum following liberation of the hernia and resection of nonviable tissue

The patient was transferred to the surgical ward with vasopressor support in the immediate postoperative period, which was successfully weaned. He was anuric during the procedure but regained adequate urine output postoperatively. Nutritional support was maintained with parenteral nutrition until oral tolerance was established. Empirical antibiotic therapy with carbapenems and metronidazole was continued pending culture results (Figure 4).

**Figure 5:**
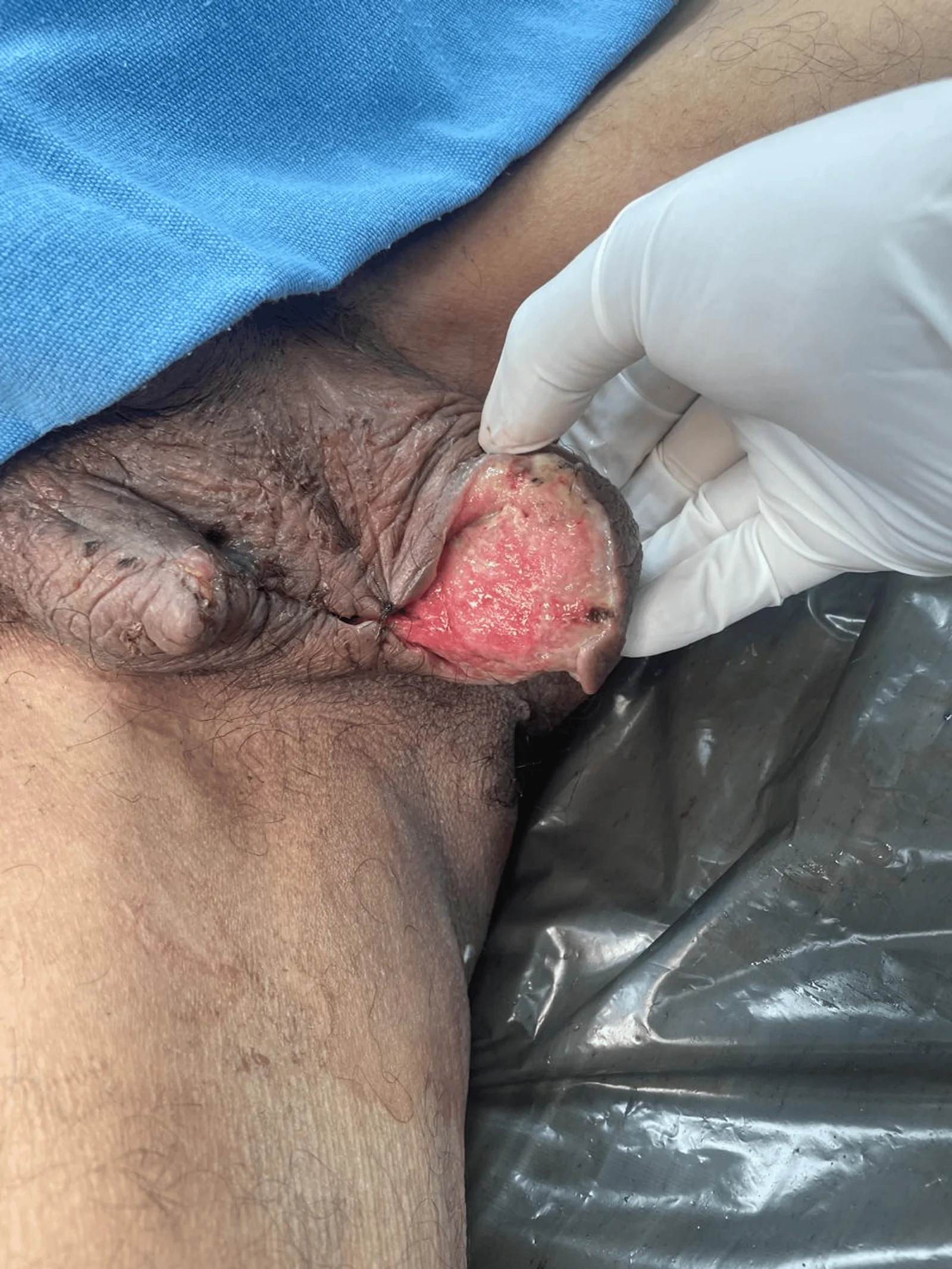
Open scrotum after five washouts

Five additional surgical washouts of the scrotal and inguinal regions were performed during the hospitalization. Wound debridement and dressing changes were performed daily. The right testis sac showed exposed tissue with early granulation, which was managed with hydrocolloid patches and sterile, soft, non-woven hydrofiber dressings. The ileostomy remained functional with controlled output. Penrose drains were removed progressively as the output decreased (Figure 5).

At discharge after 33 days of hospitalization, the patient was afebrile, with stable vital signs, a functioning ileostomy with low output, and healing scrotal wounds without evidence of active infection. He was discharged on oral antibiotics (cefixime and metronidazole), analgesics, and wound care instructions. Follow-up was scheduled at the General Surgery outpatient clinic.

During outpatient follow-up, intestinal transit restoration was performed successfully six months after discharge. The scrotum healed completely two months after the major surgery (one month after discharge). The patient is currently stable and remains under observation during scheduled follow-up appointments (Table 2).

**Table 2:** Clinical timeline Cr: Creatinine.

Date	Event	Findings/Intervention	Outcome
~5 days prior	Symptom onset	Scrotal swelling, pain, discoloration, obstipation, vomiting	No medical evaluation sought
Hospitalization day 1	Emergency admission	Inguinoscrotal mass 17 x 10 cm, necrotic base, leukocytosis 20.7k, Cr 2.4	Surgical consultation, resuscitation
Hospitalization day 2	Emergency laparotomy	30 cm approx mixed hernial content, cecal perforation, Fournier's gangrene, right testicular compromise	Bowel resection, ileostomy, orchiectomy, debridement
Hospitalization day 20	Inpatient recovery	5 scrotal washouts, use of sterile, soft, non-woven hydrofiber dressing, ileostomy functioning, drain removal	Progressive wound healing, tolerance of oral intake
Hospitalization day 33	Discharge	Stable, afebrile, low-output ileostomy, healing wounds	Oral antibiotics, outpatient follow-up
Outpatient	Intestinal transit restoration	-	Patient currently stable

## Discussion

Amyand's hernia is an anatomical rarity. In Manatakis et al.'s systematic review of 442 patients across 20 years of published literature, acute appendicitis within the hernial sac was the surgical indication in only 12% of emergency cases, and the presence of additional abdominal pathology rendering it a type 4 hernia according to the Losanoff-Basson classification was uncommon [1-4]. What further distinguishes our case is the combination of anatomical features that have not been described together in any previously indexed report: a right-sided presentation with mixed hernial contents extending 370 cm from the angle of Treitz to the scrotum, cecal rather than appendiceal perforation as the primary driver, and full-thickness scrotal involvement meeting the criteria for Fournier's gangrene.

In our patient, the length of the herniated bowel, extending from the angle of Treitz to the scrotum, suggests a long-standing mesenteric mobility that had been present for years but remained clinically silent until strangulation occurred. A 10-year history without surgical correction provides the background against which this degree of herniation becomes anatomically plausible [5,6]. Rajaguru and Ee Lee reported the only comparable case in the literature involving simultaneous gangrenous appendicitis and cecal perforation in an Amyand's hernia. That case was right-sided but did not involve Fournier's gangrene or a perforated cecum [7]. Losanoff and Basson described and later refined the classification of Amyand's hernia [3]. According to this classification, our patient presented not only with an Amyand's hernia but also with complications resulting from its long-term evolution, necessitating extensive surgery to repair the defect and manage the associated complications.

The Losanoff-Basson classification provides the surgical roadmap for Amyand's hernia. For type 2 and type 3 lesions, appendectomy with primary tissue repair without mesh is the standard approach [3,4]. For type 4 lesions, the classification recommends investigation and treatment of the concurrent abdominal pathology together with hernia repair, while explicitly avoiding prosthetic mesh in contaminated fields [4,5]. We followed this approach. The contaminated operative field caused by cecal perforation and fecaloid spillage precluded mesh placement, and primary closure of the hernia defect was performed using endogenous tissue repair. This decision is consistent with current recommendations indicating that mesh avoidance in heavily contaminated Amyand's hernias does not increase recurrence when primary repair achieves adequate tension-free closure [8,9].

Fournier's gangrene complicating Amyand's hernia is itself a rarely reported entity. Rajaguru and Ee Lee described a type 4 Amyand's hernia complicated by Fournier's gangrene in a 47-year-old male, in whom the diagnosis was established intraoperatively and the source of contamination was a perforated appendix within the inguinal sac [8]. Our case shares the intraoperative discovery and the type 4 classification but differs in the source of contamination, the extent of bowel involvement, and the requirement for multivisceral resection, including orchiectomy. Fournier's gangrene carries a mortality rate exceeding 20% in most series, with the highest disease-related deaths occurring within the first seven days [6,7]. Aggressive source control, early debridement, and broad-spectrum antibiotic coverage remain the cornerstones of management [6,9].

The decision to perform a right orchiectomy was based on intraoperative findings of testicular compromise with no viable tissue. Low-volume centers managing Fournier's gangrene have higher orchiectomy rates than high-volume centers, reflecting the resource constraints that may shape surgical decision-making in settings similar to ours [6]. The five additional scrotal washouts performed during hospitalization, combined with the use of sterile, soft, non-woven hydrofiber dressings to the exposed left testis, represented a staged approach to wound management that prioritized infection control before definitive reconstruction. This strategy is supported by current evidence favoring delayed primary closure over immediate reconstruction in Fournier's gangrene, with favorable outcome series reporting median debridement counts of two to three procedures before wound closure [9].

Intestinal transit restoration was planned from the outset as a deferred procedure. Accordingly, a terminal ileostomy with a closed colonic pouch was created instead of performing a primary anastomosis because of the degree of fecal contamination, the patient's hemodynamic instability requiring vasopressor support in the immediate postoperative period, and the nutritional compromise reflected by a serum albumin concentration of 2.9 g/dL at admission. These factors are recognized contraindications to primary anastomosis in emergency colorectal surgery [10,11]. The subsequent successful outpatient restoration of intestinal transit confirmed that the staged approach was both safe and effective in this context.

A 10-year delay in hernia repair created the anatomical conditions for this presentation. Rural populations with limited access to elective surgical care are disproportionately affected by this pattern, in which watchful waiting becomes the default because of circumstance rather than clinical choice. The clinical lesson is direct: an inguinoscrotal hernia with skin discoloration and inability to reduce in a patient with symptoms of bowel obstruction requires emergency surgical evaluation, regardless of the duration of the hernia history, as the degree of visceral herniation and the risk of Fournier's gangrene increase over time. Preoperative CT, when the patient's hemodynamic status allows, can define the hernial content and its extent before incision, potentially guiding the operative approach. Although primary hernia repair is normally performed using mesh, in this case the operative field was considered high risk. Therefore, primary closure under tension was performed despite the high risk of hernia recurrence, which may be as high as 30%. The primary objective of this intervention was to alleviate the pressure on the herniated bowel and stop the fecal leakage. If the hernia recurs, it will be addressed at a later stage [10,11].

## Conclusions

This case documents a right-sided Amyand's hernia with mixed cecal, appendiceal, and ileal contents extending 370 cm into the scrotum, complicated by cecal perforation and Fournier's gangrene, and managed with emergency multivisceral resection, colonic pouch formation, terminal ileostomy, right orchiectomy, staged scrotal debridement, and subsequent intestinal transit restoration. To our knowledge, it represents the most anatomically extensive right-sided Amyand's hernia with simultaneous cecal perforation and Fournier's gangrene reported in the indexed literature.​​​​​

Three practical points emerge from this case. First, right-sided Amyand's hernia, although accounting for fewer than 10% of all Amyand's hernias, should be considered when a right inguinoscrotal hernia presents with signs of strangulation and bowel obstruction, as the surgical complexity exceeds that of a standard incarcerated hernia. Second, cecal perforation, and not only appendiceal perforation, can be the source of Fournier's gangrene in the context of a strangulated Amyand's hernia, and the operative plan must account for multivisceral involvement from the outset. Third, a staged approach, with source control and diversion performed first and reconstruction later, is both safe and achievable even in resource-limited settings when surgical decision-making is timely. The prolonged untreated hernia history in this patient underscores the ongoing need for accessible elective surgical care in rural populations, as well as improved public awareness of the potential complications of untreated hernias. It also highlights the importance of educating patients about the optimal timing of surgical repair and the potentially life-threatening complications that may arise from the natural progression of hernias, as illustrated by the giant inguinoscrotal hernia presented in this case.
